# Composite Probiotic Fermented Feed Enhances Growth Performance and Intestinal Health in Weaned Piglets by Modulating the Gut Microbiome and Metabolome

**DOI:** 10.3390/ani16060972

**Published:** 2026-03-20

**Authors:** Zifan Wang, Zhimin Lin, Binbin Lin, Song Peng, Yijuan Xu, Xiuzhen Wang, Huini Wu, Bilin Xie, Bihong Chen, Mengshi Zhao, Fengqiang Lin, Tiecheng Sun, Zhaolong Li

**Affiliations:** 1 Institute of Animal Husbandry and Veterinary Medicine, Fujian Academy of Agricultural Sciences, Fuzhou 350013, China; w_zifan2022@163.com (Z.W.);; 2 Putian Agricultural Science Research Institute, Putian 351100, China; 3 Institute of Subtropical Agriculture, Fujian Academy of Agricultural Sciences, Zhangzhou 363005, China; 4 Quanzhou Animal Husbandry Station, Quanzhou 362000, China; 5 Fujian Provincial Animal Husbandry Station, Fuzhou 350001, China

**Keywords:** nursery pig, growth performance, intestinal health, gut microbiota, metabolites

## Abstract

This study investigated the effects of compound microbial fermented feed on the growth performance, intestinal architecture, microbiota composition, and metabolic profiles of weaned piglets. Fifty-four weaned piglets were randomly allocated to three dietary treatment groups: a control group (basal diet), a 50% fermented feed group (T1), and a 100% fermented feed group (T2), for a 33-day feeding period. The results indicated that both T1 and T2 diets significantly improved final body weight and average daily gain (ADG), while decreasing the feed-to-gain ratio (F/G) compared with the control (*p* < 0.05). Morphological assessment revealed that the T1 group significantly elevated the villus height-to-crypt depth ratio in the jejunum and increased the density of goblet cells in the cecum and colon (*p* < 0.05). Multi-omics analysis indicated that fermented feed significantly reshaped the gut microbiota structure (*p* < 0.05), characterized by the enrichment of beneficial taxa, including *Oscillospiraceae* and *Lachnospiraceae* (*p* < 0.05), and the modulation of nucleotide and bile acid metabolism. Furthermore, correlation analysis identified significant linkages between the abundance of jejunal *Oscillospiraceae* and colonic/cecal *Lactobacillus* with growth performance, intestinal morphology, and key metabolites. This finding systematically elucidates the mechanisms by which compound microbial fermented feed promotes growth and intestinal health in weaned piglets via microbiota-mediated pathways, offering a robust scientific framework for the development of antibiotic-free nutritional strategies.

## Simple Summary

Weaning imposes significant physiological stress on piglets, often impairing growth and intestinal health. Here, we investigate whether dietary intervention with a compound microbial fermented feed can mitigate these effects. Over a 33-day period, weaned piglets were fed either a standard diet or diets in which 50% or 100% of the feed was replaced with the fermented formulation. Both fermented feed groups exhibited accelerated growth and improved feed efficiency. Notably, the 50% fermented feed group also displayed enhanced intestinal architecture and an increased abundance of beneficial bacteria, including *Lachnospiraceae*. Multi-omics and correlation analyses directly linked these microbial shifts to the observed improvements in growth performance and health markers. Our findings provide robust evidence that compound microbial fermented feed promotes piglet health and performance through microbiota-mediated mechanisms, offering a scientific basis for the development of effective antibiotic-free nutritional strategies in sustainable livestock production.

## 1. Introduction

The global restriction of antibiotic growth promoters in livestock production has necessitated the identification of safe and effective alternatives [1]. Compound microbial fermented feed (CMFF), characterized by a rich profile of probiotics, prebiotics, and bioactive metabolites, has emerged as a promising strategy for modulating gut homeostasis and sustaining growth performance. The nursery phase in swine production is particularly critical for the implementation of antibiotic-free technologies, as weaning stress frequently induces gut microbiota dysbiosis, compromises barrier integrity, and impairs growth [2,3,4]. Consequently, elucidating how CMFF systematically remodels the gut microecology to enhance host health and productivity is of substantial theoretical and practical importance for the sustainable transition of animal husbandry.

Previous studies have demonstrated that probiotic-fermented feed improves growth performance and intestinal health in nursery pigs [5,6,7,8]. Specifically, fermented feeds containing lactic acid bacteria have been shown to increase average daily gain and reduce the incidence of diarrhea [6,9]. Morphologically, such interventions elevate the villus height-to-crypt depth ratio and bolster mucosal immune function [7,10]. Furthermore, fermented feed and its associated metabolites modulate the gut microbiota by enriching beneficial short-chain fatty acid-producing taxa, such as *Lachnospiraceae* and *Ruminococcaceae*, while suppressing potential pathogens like Campylobacter. Recent advancements in metabolomics indicate that fermented feed profoundly influences host metabolic status through microbial activity; for instance, probiotic interventions modulate bile acid profiles and nucleotide metabolism, which are directly involved in nutrient absorption, immune regulation, and epithelial renewal [6,11,12,13,14]. Despite these insights, existing research has primarily focused on partially fermented diets, leaving the physiological impacts of fully fermented diets largely unexplored [6,9,12,13,14,15].

Here, we hypothesized that complete-diet fermentation improves growth performance and intestinal health in nursery pigs by modulating the gut microbiota and their associated metabolites and that these effects are dose-dependent. To test this hypothesis, we employed an integrated multi-omics approach in a 33-day feeding trial. A total of 28-day-old nursery pigs were randomly assigned to three dietary treatments: a basal diet (control), a diet containing 50% fermented feed (T1), and a diet containing 100% fermented feed (T2). By integrating 16S rDNA high-throughput sequencing with untargeted metabolomics, we systematically evaluated the impact of varying fermented feed inclusion levels on growth performance, intestinal morphology (jejunum, cecum, and colon), and luminal microbial-metabolite profiles. Finally, we constructed microbial-metabolite-host association networks via Spearman correlation analysis to pinpoint the key mechanisms driving the observed improvements in growth and intestinal health.

## 2. Materials and Methods

### 2.1. Experimental Animals and Design

This study utilized a total of 54 healthy nursery pigs (Duroc × Landrace × Yorkshire crossbred), aged 28 days, with an average body weight of 11.94 ± 0.36 kg. The piglets were sourced from a commercial pig farm located in Putian City, China. All nursery pigs were housed within the same building, with separate pens that were maintained under fully enclosed environmental control. Throughout the experimental period, the piglets had ad libitum access to both feed and water. Routine farm management procedures, including barn disinfection, deworming, and vaccination protocols, were strictly adhered to for all animals. All animal procedures were approved by the Animal Ethics Committee of the Institute of Animal Husbandry and Veterinary Medicine, Fujian Academy of Agricultural Sciences (approval No. [MYLISC2025-084]) and were conducted in accordance with the relevant ethical guidelines. The piglets were randomly assigned to three dietary groups (balanced for both body weight and sex), with three replicates (per pen) per group and six piglets per replicate (per pen). Each pen contained three females and three males to ensure consistent sex distribution across replicates. The control group (C1) was fed a basal diet, while the two treatment groups received diets in which 50% (T1 group) or 100% (T2 group) of the basal diet was replaced with probiotic-fermented feed. The trial included a 3-day adaptation period, followed by a 30-day formal feeding period. The pen size is 2.5 m × 2.0 m = 5.0 m^2^, with nipple-type waterers and feed troughs, and the ambient temperature is 26~28 °C, and the relative humidity is 60~70%.

### 2.2. Experimental Diets and Fermentation Strains

A corn–soybean meal-based diet, formulated as mash feed to meet the nutritional requirements for pigs weighing 11–25 kg according to NRC (2012) standards [16], served as the basal diet. The formulations and nutritional compositions of both the control diet and the fermented feeds are provided in Table 1. The fermentation process employed pig-derived probiotics, specifically *Bacillus subtilis strain* (BSS-37) and *lactic acid bacteria* (LAB-68) (these strains were isolated from the jejunal mucosa of piglets; based on their morphological, physiological, and biochemical characteristics, as well as 16S rRNA gene sequence analysis, we conducted a comprehensive evaluation) and was conducted in four sequential steps: First, strain expansion and activation involved the sequential propagation of preserved strains (from agar slant to liquid or solid culture in conical flasks, and then to production-scale seed culture or broth) under sterile or clean conditions, with controlled medium, temperature (approximately 30 °C), and time to restore high activity. Second, raw material pretreatment and mixing involved proportionally mixing feed ingredients and adjusting the moisture content to 20–30% to create a suitable initial environment for microbial growth. Third, inoculation and phased fermentation control—the core procedure—utilized an “aerobic followed by anaerobic” strategy. In the aerobic phase, activated strains were inoculated and thoroughly mixed into the material, which was moderately aerated and maintained at 30–35 °C for 1–3 days to promote the rapid growth of aerobic and facultative anaerobic bacteria, macromolecular decomposition, and heat generation. When the temperature peaked (>50 °C) and began to decline, the material was transferred to sealed containers or compacted to initiate the anaerobic phase, dominated by lactic acid bacteria at 30–37 °C for 5–7 days or longer, primarily aimed at acid production, flavor compound accumulation, and nutrient preservation. Finally, fermentation termination, drying, and storage were executed: fermentation was deemed complete when a strong, savory, and acidic aroma developed and the pH stabilized at a low level (<4.5). The process was immediately terminated by low-temperature drying (≤50 °C) or air-drying to reduce moisture below 10%, thus preventing spoilage; the final product was then ground, packaged, and stored in a cool, dry place.

### 2.3. Sample Collection

The animal experimental protocol received approval from the Animal Ethics Committee of the Institute of Animal Husbandry and Veterinary Medicine, Fujian Academy of Agricultural Sciences (approval No. MYLISC2025-084). All procedures were conducted in strict compliance with international animal welfare guidelines (ARRIVE guidelines) and relevant national regulations, with efforts made to minimize the number of animals used and to alleviate suffering. On day 30 of the trial, three piglets were randomly selected from each group and euthanized via intravenous injection of an overdose of pentobarbital sodium. Following euthanasia, the thoracic and abdominal cavities were opened immediately. Each intestinal segment was ligated, and the luminal contents from corresponding sites of the jejunum (K), cecum (M), and colon (J) were aseptically collected. The samples were placed into sterile 2 mL cryovials, rapidly frozen in liquid nitrogen, and subsequently transferred to a −80 °C freezer for long-term storage. These samples were intended for subsequent 16S rDNA high-throughput sequencing of the gut microbiota and untargeted metabolomic analysis. The entire sampling procedure was conducted either on ice or with expedited handling to ensure sample integrity.

For feed analysis, representative samples of the experimental diets (C1, T1, and T2) were collected at both the beginning and the end of the trial. Approximately 500 g of each diet was randomly sampled from multiple locations within each feed batch, pooled to form a composite sample per diet, and stored in sealed plastic bags at −20 °C until subsequent chemical analysis. The samples were analyzed for dry matter (DM; AOAC Official Method 930.15), crude protein (CP; AOAC Official Method 984.13), crude fat (AOAC Official Method 920.39), crude fiber (AOAC Official Method 978.10), and ash (AOAC Official Method 942.05). Gross energy was determined using an adiabatic bomb calorimeter (C200, IKA, Staufen, Germany). DM was measured by oven-drying at 105 °C for 24 h in a forced-air drying oven (UFE 500, Memmert, Schwabach, Germany). CP was analyzed by the Kjeldahl method using a Kjeltec 8400 analyzer (FOSS, Hillerød, Denmark). Crude fat was extracted with petroleum ether using a Soxhlet apparatus (SER 148, VELP Scientifica, Usmate, Italy). Crude fiber was determined with a fiber analyzer (FIWE 6, VELP Scientifica, Usmate, Italy). Ash content was obtained by incineration in a muffle furnace (L 9/11, Nabertherm, Lilienthal, Germany) at 550 °C for 6 h. All analyses were performed in triplicate, and the nutrient composition presented in Table 1 (determined nutrient levels) includes gross energy, crude protein, ether extract, neutral detergent fiber, acid detergent fiber, ash, calcium, total phosphorus, and standardized ileal digestible amino acids.

### 2.4. Methods

#### 2.4.1. Measurement of Growth Performance

At the beginning and end of the trial, all piglets were individually weighed after an overnight fast to record their initial body weight (IBW) and final body weight (FBW), respectively. The average daily gain (ADG) was calculated using the following formula: (FBW − IBW)/number of feeding days. The total feed supplied and the residual feed per pen were recorded daily to compute the average daily feed intake (ADFI). The feed-to-gain ratio (F/G) was determined by dividing ADFI by ADG.

#### 2.4.2. Morphological Observation of the Jejunum, Cecum, and Colon

Segments measuring 1–2 cm in length from the jejunum, cecum, and colon were collected from corresponding anatomical locations in each group. After gentle rinsing with physiological saline, the tissues were fixed in 4% paraformaldehyde for 48 h. The fixed tissues were subsequently dehydrated, embedded in paraffin, sectioned, and mounted on slides to prepare paraffin sections for hematoxylin–eosin (HE) and Alcian Blue–Periodic Acid Schiff (AB-PAS) staining. HE Staining Procedure: Paraffin sections were deparaffinized using xylene and rehydrated. They were then stained with hematoxylin solution, differentiated with acidic alcohol, blued with ammonia solution, counterstained with eosin, dehydrated through a graded ethanol series, cleared in xylene, and finally mounted for observation. AB-PAS Staining Procedure: Following deparaffinization and rehydration, sections were stained with Alcian Blue solution, oxidized with an oxidizing agent, and subsequently stained with Schiff’s reagent. The sections were then counterstained with hematoxylin, dehydrated through a graded ethanol series, cleared in xylene, and mounted. All stained sections were scanned using a PANNORAMIC panoramic slide scanner (3DHISTECH Ltd., Budapest, Hungary). The scanned images were viewed and assessed under 40× (for HE) or 100× (for AB-PAS) magnification using CaseViewer 2.4 software. Image analysis was conducted using Image-Pro Plus 6.0 software (Media Cybernetics, Rockville, MD, USA) to measure jejunal villus height (VH) and crypt depth (CD), as well as the number of goblet cells and the corresponding epithelial length in the cecum and colon. The villus height-to-crypt depth ratio (VH/CD) and the number of goblet cells per unit length of epithelium (goblet cell count/epithelial length) were calculated.

#### 2.4.3. Determination of Microbial Communities in the Jejunum, Cecum, and Colon of Nursery Pigs

The intestinal contents collected from the jejunum, cecum, and colon in sterile cryovials were used for total genomic DNA extraction employing a bacterial DNA extraction kit. The extracted DNA served as the template for PCR amplification of the bacterial 16S rRNA V3–V4 hypervariable region, utilizing the primers 341F (5′-CCTACGGGNGGCWGCAG-3′) and 805R (5′-GACTACHVGGGTATCTAATCC-3′). Sequencing libraries were constructed using the NEBNext^®^ Ultra™ II DNA Library Prep Kit (New England Biolabs, Ipswich, MA, USA) and subsequently assessed for quality. Qualified libraries were sequenced on the Illumina NovaSeq 6000 platform (Illumina, San Diego, CA, USA). The raw paired-end reads (RawData) were merged based on their overlapping regions, followed by filtering out low-quality sequences and chimeras to obtain high-quality clean data (CleanData). The DADA2 plugin in QIIME2 was employed for sequence denoising, generating representative amplicon sequence variants (ASVs), and an ASV abundance table. These outputs were utilized for subsequent downstream analyses, including alpha and beta diversity analysis, linear discriminant analysis effect size (LEfSe), and functional prediction.

#### 2.4.4. Untargeted Metabolomic Profiling of Intestinal Contents from Jejunum, Cecum, and Colon in Nursery Pigs

The analysis of intestinal content samples collected from the jejunum, cecum, and colon was conducted using LC-MS/MS. The primary procedures involved homogenizing approximately 25 ± 1 mg of each sample with grinding beads and 500 μL of extraction solvent (methanol:acetonitrile:water, 2:2:1, *v*/*v*) containing isotope-labeled internal standards, followed by vortexing for 30 s. Chromatographic separation was achieved using an ultra-high-performance liquid chromatography system (Vanquish, Thermo Fisher Scientific, Waltham, MA, USA), equipped with a Waters ACQUITY UPLC BEH Amide column (2.1 mm × 50 mm, 1.7 μm) and coupled to an Orbitrap Exploris 120 mass spectrometer (Thermo Fisher Scientific). The mobile phase consisted of 25 mmol/L ammonium acetate and 25 mmol/L ammonium hydroxide in water (A) and acetonitrile (B). The autosampler temperature was maintained at 4 °C, and the injection volume was set at 2 μL. The Orbitrap Exploris 120 mass spectrometer, controlled by Xcalibur software (version 4.4, Thermo), was operated to acquire both MS^1^ and MS/MS data. The detailed parameters were as follows: sheath gas flow of 50 Arb; auxiliary gas flow of 15 Arb; capillary temperature of 320 °C; full-scan MS resolution of 60,000; MS/MS resolution of 15,000; collision energy set to stepped NCE 20/30/40; and spray voltage of 3.8 kV for positive ion mode and −3.4 kV for negative ion mode. The raw data files were converted to mzXML format using ProteoWizard software (V3.0.X) and subsequently processed with an in-house R script based on XCMS for peak detection, extraction, alignment, and integration. Metabolite identification was performed using R packages (V4.9.1) in conjunction with the BiotreeDB (V3.0) database.

### 2.5. Data Processing and Analysis

Data on growth performance (n = 18) and histological measurements (n = 6) were analyzed using one-way analysis of variance (ANOVA) in SPSS version 26.0. When ANOVA indicated a significant overall effect (*p* < 0.05), Tukey’s honestly significant difference (HSD) test was employed as a post hoc multiple comparison test to assess differences among the three dietary groups (C1, T1, and T2). Results are presented as mean ± standard deviation (SD). Differences were deemed statistically significant at *p* < 0.05 and non-significant at *p* > 0.05. The significance markers (superscript letters a, b, c) presented in the Results Section (Table 2 and Table 3 and associated figures) were assigned based on the outcomes of this Tukey’s HSD test. Preprocessed data from microbiome and metabolomics analyses (n = 6) were uploaded to the OmicsStudio Cloud Platform (https://www.omicstudio.cn/tool (accessed on 12 November 2025)) for statistical analysis and visualization. For microbiome data, analyses of alpha diversity, beta diversity, and linear discriminant analysis effect size (LEfSe) were conducted. Beta diversity was assessed using principal coordinate analysis (PCoA) based on Bray–Curtis distances. To statistically evaluate the differences in microbial community structure among the dietary groups (C1, T1, and T2), permutational multivariate analysis of variance (PERMANOVA, also known as Adonis) was performed with 999 permutations using the “vegan” package in R. The resulting R^2^ and *p* values are reported to indicate the effect size and significance of group separation. For metabolomics data (n = 6), principal component analysis (PCA) and orthogonal partial least squares—discriminant analysis (OPLS-DA) models were constructed to derive Variable Importance in the Projection (VIP) scores. Differential metabolites between groups were identified based on the following criteria: VIP > 1, fold change (FC) > 2 or < 0.5, and *p* < 0.05. KEGG pathway enrichment analysis of the identified metabolites was performed using the Biotree Lims2 Platform (https://biotree.lims2.com (accessed on 3 December 2025)). Spearman correlation analysis was conducted and visualized to assess relationships among the feed-to-gain ratio, villus height, villus height-to-crypt depth ratio, the number of goblet cells per unit length, key microbial taxa, and significant differential metabolites.

## 3. Results

### 3.1. Effects of Compound Microbial Fermented Feed on Growth Performance

As presented in Table 2, the final body weight of nursery pigs in the T1 and T2 groups was significantly greater than that of the C1 group (*p* < 0.05). The average daily gain (ADG) was markedly higher in the T2 group (*p* < 0.05), whereas no significant difference was noted in the T1 group (*p* > 0.05). Additionally, both the T1 and T2 groups exhibited significantly reduced feed-to-gain ratio (F/G) (*p* < 0.05). These findings suggest that the diets in the T1 and T2 groups effectively enhanced the growth performance of nursery pigs.

### 3.2. Effects of Compound Microbial Fermented Feed on Intestinal Morphology

**Figure 1:**
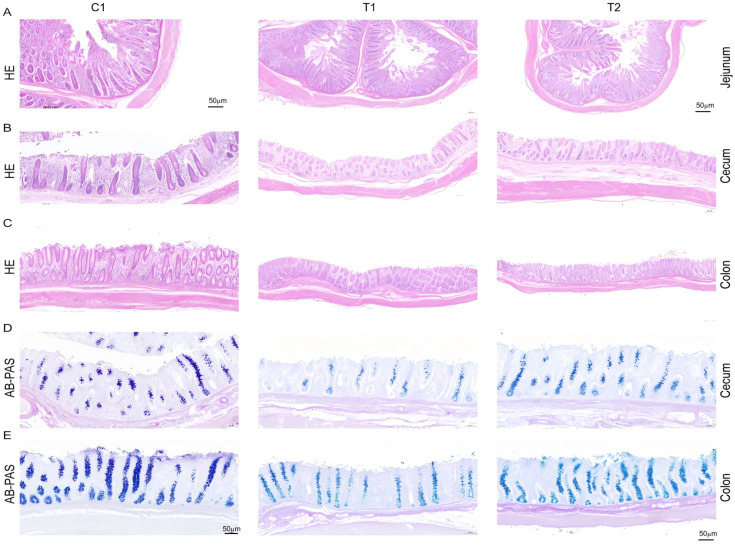
Effects of compound microbial fermented feed on intestinal morphology of nursery pigs ((**A**): jejunum HE staining (40×). (**B**): cecum HE staining (40×). (**C**): colon HE staining (40×). (**D**): cecum AB-PAS staining (100×). (**E**): colon AB-PAS staining (100×)).

As presented in Table 3, the villus height-to-crypt depth ratio in the jejunum of nursery pigs in the T1 group was significantly increased by 37.5% compared to the C1 group (*p* < 0.05). No significant differences were observed in villus height, crypt depth, or the villus height-to-crypt depth ratio in the jejunum of the T2 group (*p* > 0.05). In the cecum, both the T1 and T2 groups demonstrated significant increases in the number of goblet cells by 115.2% and 131.7%, respectively, and epithelial length by 104.4% and 100.0% (*p* < 0.05); however, no significant difference was noted in the number of goblet cells per unit length (*p* > 0.05). In the colon, the T1 group exhibited significant increases in the number of goblet cells by 101.0%, in epithelial length by 59.3%, and in the number of goblet cells per unit length (*p* < 0.05). The T2 group showed a significant increase in epithelial length by 25.7%, compared to the C1 group (*p* < 0.05), but no significant differences were found in the number of goblet cells or the number of goblet cells per unit length (*p* > 0.05). Representative HE-stained sections of the jejunum are illustrated in Figure 1A, while AB-PAS-stained sections of the cecum and colon are depicted in Figure 1D and Figure 1E, respectively.

### 3.3. Effects of Compound Microbial Fermented Feed on Gut Microbiota

#### 3.3.1. Venn Diagram of ASV Distribution, and Alpha Diversity Analyses

**Figure 2:**
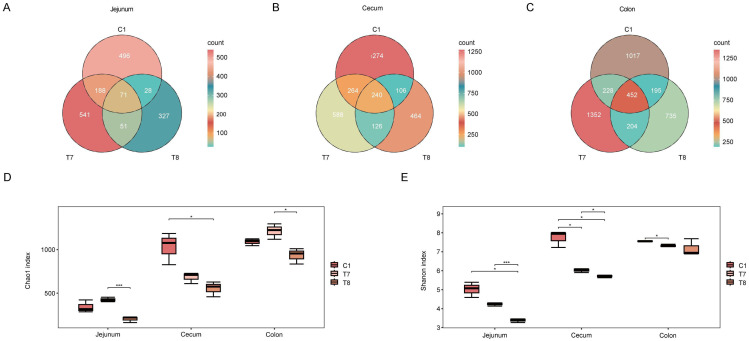
Effects of different treatments on microbial communities across intestinal segments in nursery pigs. The distribution of amplicon sequence variants (ASVs) in the jejunum, cecum, and colon is shown in panels (**A**), (**B**), and (**C**), respectively. Alpha diversity metrics, including the Chao1 richness estimator (**D**) and Shannon diversity index (**E**), are presented for all three intestinal segments (* *p* < 0.05; *** *p* < 0.0001).

The effects of various treatments on the microbial communities within each intestinal segment of nursery pigs were assessed by analyzing ASV distribution (Figure 2A–C), alpha diversity (Figure 2D,E). ASV analysis indicated that, compared to the control group (C1), the T1 treatment exhibited an increasing trend in the number of ASVs in both the jejunum and colon, whereas the T2 treatment displayed a decreasing trend across all intestinal segments. Alpha diversity analysis revealed that the effects of the treatments were specific to each segment. In the jejunum, the T2 treatment significantly decreased the Shannon index (*p* < 0.05, Figure 2E) but did not significantly affect richness. In the cecum, both T1 and T2 treatments significantly reduced the Shannon index (*p* < 0.05), with the T2 treatment also significantly lowering the Chao1 index (*p* < 0.05, Figure 2D). In the colon, only the T1 treatment significantly decreased the Shannon index (*p* < 0.05, Figure 2E). 

#### 3.3.2. Analysis of Microbial Composition at Phylum and Genus Levels

**Figure 3:**
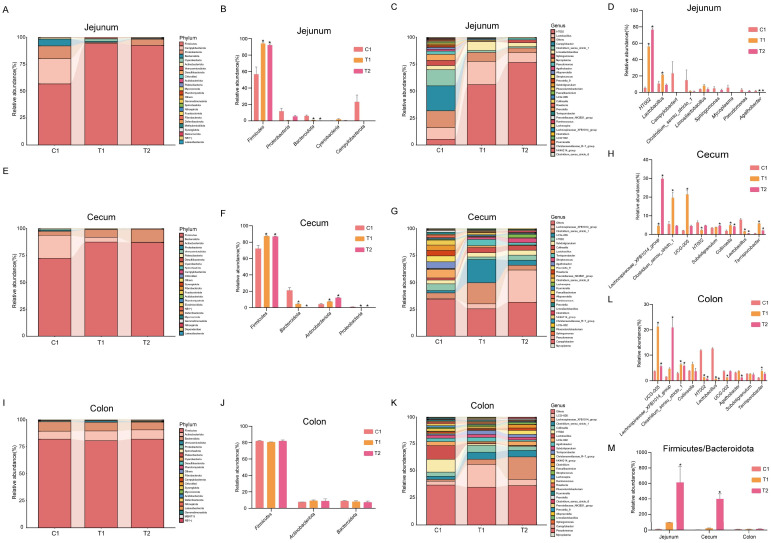
Changes in the colonic microbiota composition at the phylum and genus levels, and the Firmicutes/Bacteroidetes (F/B) ratio. (**A**,**B**) Jejunum—phylum level; (**C**,**D**) jejunum—genus level; (**E**,**F**) cecum—phylum level; (**G**,**H**) cecum—genus level; (**I**,**J**) colon—phylum level; (**K**,**L**) colon—genus level; and (**M**) ratio of Firmicutes to Bacteroidetes (F/B) in the jejunum, cecum, and colon across the three dietary groups (C1, T1, T2). Data are presented as mean ± SEM (n = 6). “*” indicates significant differences between groups (*p* < 0.05), as determined by one-way ANOVA followed by Tukey’s HSD post hoc test.

At the phylum level, the jejunal microbiota was predominantly composed of *Firmicutes*, *Proteobacteria*, and *Bacteroidetes*. Compared to the C1 group (n = 6), both the T1 and T2 groups (n = 6) exhibited a significant increase in *Firmicutes* and a notable decrease in *Bacteroidetes*. Furthermore, a reduction in potentially harmful Campylobacterota was observed in these treatment groups (Figure 3A,B). At the genus level, the primary taxa included HT002, *Lactobacillus*, and *Campylobacter*. Relative to the C1 group, HT002 significantly increased in both the T1 and T2 groups, whereas *Alloprevotella* showed a significant decrease. The T1 group also exhibited a marked increase in Lactobacillus. Both treatment groups demonstrated a reduction in potentially harmful genera such as *Campylobacter* and *Mycoplasma* (Figure 3C,D). In the cecum, the dominant phyla were *Firmicutes, Bacteroidetes*, and *Actinobacteriota*. Compared to the C1 group, both the T1 and T2 groups showed a significant increase in *Firmicutes* and *Actinobacteriota*, accompanied by a significant decrease in *Bacteroidetes* and *Proteobacteria* (Figure 3E,F). At the genus level, the main taxa included *Lachnospiraceae*_XPB1014_group, *Clostridium*, and UCG-005. Compared to the C1 group, both treatment groups exhibited significant increases in *Lachnospiraceae*_XPB1014_group, *Collinsella*, and *Terrisporobacter*, while *Lactobacillus* significantly decreased. Additionally, the T1 group showed significant increases in *Clostridium* and UCG-005 and a significant decrease in HT002. The T2 group exhibited a significant increase in Subdoligranulum (Figure 3G,H). The colonic microbiota was primarily dominated by *Firmicutes*, *Actinobacteriota*, and *Bacteroidetes* at the phylum level, with no significant differences observed among treatment groups (Figure 3I,J). At the genus level, dominant taxa included UCG-005, *Lachnospiraceae*_XPB1014_group, and *Clostridium*. Compared to the C1 group, both the T1 and T2 groups showed significant increases in UCG-005 and *Clostridium*, alongside significant decreases in HT002 and *Lactobacillus*. The T1 group exhibited a significant decrease in UCG-002 and a significant increase in *Terrisporobacter*, while the T2 group demonstrated a significant increase in *Lachnospiraceae*_XPB1014_group and a significant decrease in *Alloprevotella* (Figure 3K,L). Analysis of the *Firmicutes*-to-*Bacteroidetes* (F/B) ratio revealed that, compared to the C1 group, the T2 group showed a significant increase in this ratio in both the jejunum and cecum. The T1 group displayed an increasing trend in the F/B ratio in these two segments, although this change was not statistically significant. No significant changes in the F/B ratio were observed in the colon for either treatment group (Figure 3M).

#### 3.3.3. Analysis of Differential Taxa

**Figure 4:**
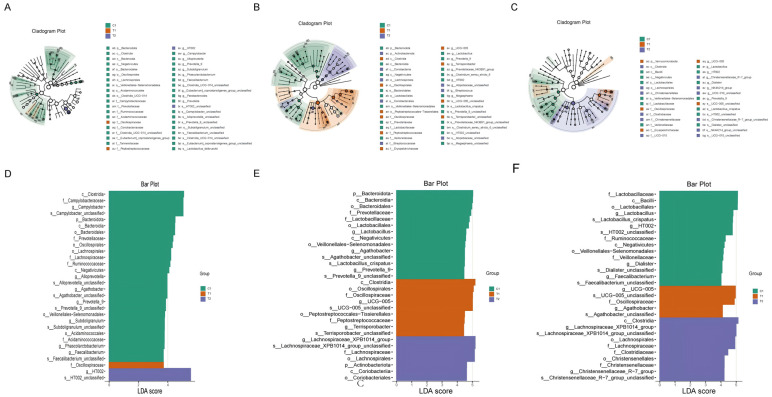
Linear discriminant analysis effect size (LEfSe) identifies differentially abundant microbial taxa among the three dietary groups (C1, T1, T2) in different intestinal segments. (**A**) Cladogram representing the taxonomic distribution of enriched taxa in the jejunum. (**B**) Cladogram for the cecum. (**C**) Cladogram for the colon. (**D**) Bar plot showing the linear discriminant analysis (LDA) scores of differentially abundant taxa in the jejunum (LDA threshold > 2, *p* < 0.05). (**E**) Bar plot for the cecum (LDA threshold > 4, *p* < 0.05). (**F**) Bar plot for the colon (LDA threshold > 2, *p* < 0.05). Only taxa meeting the significance criteria are displayed.

Based on LEfSe analysis, characteristic microbial taxa were identified as enriched in different intestinal segments across treatment groups (Figure 4). In the jejunum (LDA > 2; Figure 4A,D), the signature taxa for the T1 group were *Oscillospiraceae*, while for the T2 group it was g_HT002. The control group (C1) was enriched in multiple taxa, including *Clostridia*, *Bacteroidota*, *Lachnospiraceae*, and *Ruminococcaceae*. In the cecum (LDA > 4; Figure 4B,E), the T1 group primarily showed enrichment of taxa related to *Clostridia*, *Oscillospiraceae*, and g_UCG-005. The dominant taxa in the T2 group included the *Lachnospiraceae*_XPB1014_group and related taxa, as well as *Coriobacteriia* and its corresponding order under the phylum *Actinobacteriota*. The control group was enriched in *Bacteroidota*, *Lactobacillus*, *Agathobacter*, and *Lactobacillus crispatus*, among others. In the colon (Figure 4C,F), the signature taxa for the T1 group included g_UCG-005, *Oscillospiraceae*, and *Agathobacter*. The T2 group was mainly enriched in various taxa belonging to *Clostridia*, *Lachnospiraceae*, and *Clostridiaceae*. The control group was characterized by enrichment of *Lactobacillus*, *g_HT002*, *Ruminococcaceae*, and *Negativicutes*.

### 3.4. Effects of Compound Microbial Fermented Feed on Intestinal Metabolites

#### 3.4.1. Classification Analysis of Metabolites

LC-MS/MS analysis identified a total of 2941 compounds in the jejunum, cecum, and colon. The major classes of these metabolites included: lipids and lipid-like molecules (22.78%); organic acids and derivatives (18.77%); organoheterocyclic compounds (18.23%); benzenoids (10.10%); organic oxygen compounds (5.58%); phenylpropanoids and polyketides (5.24%); nucleosides, nucleotides, and analogs (2.24%); organic nitrogen compounds (2.14%); and alkaloids and derivatives (1.22%); among others.

#### 3.4.2. Principal Component Analysis and Orthogonal Partial Least Squares–Discriminant Analysis

**Figure 5:**
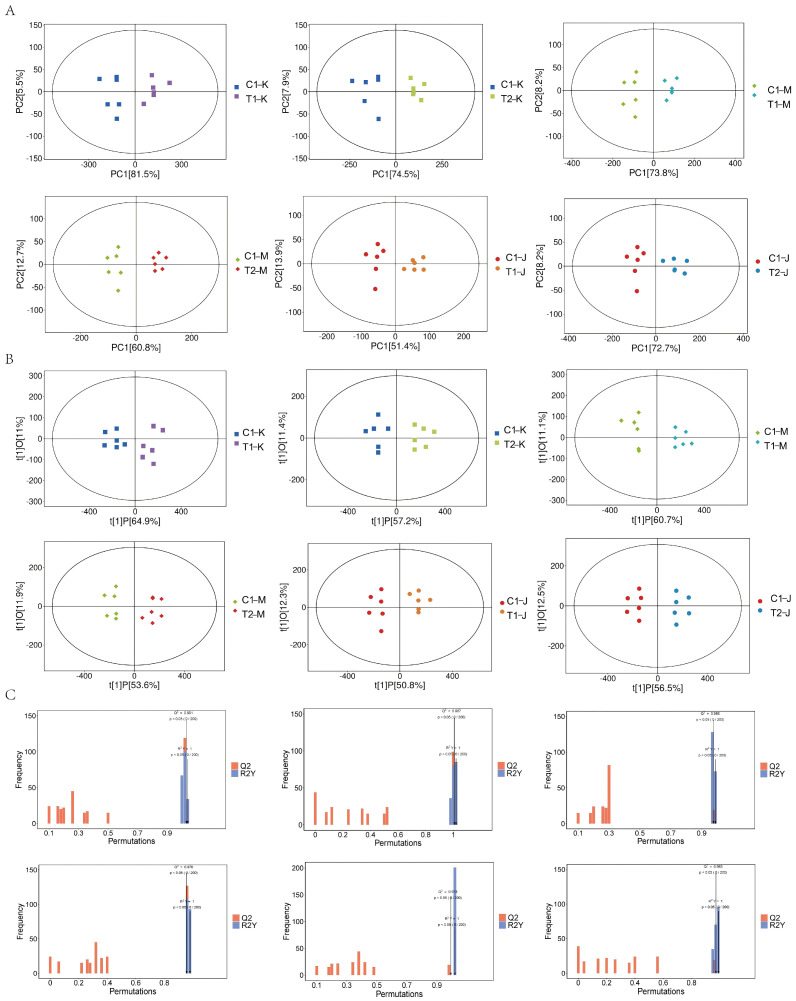
Multivariate statistical analysis of metabolomic data from jejunal, cecal, and colonic contents of weaned piglets fed different experimental diets (C1, T1, and T2). (**A**) Principal component analysis (PCA) score scatter plot showing the overall distribution and clustering of samples from the three intestinal segments across the dietary groups. Each point represents an individual sample, and ellipses indicate 95% confidence intervals. (**B**) Orthogonal partial least squares–discriminant analysis (OPLS-DA) score scatter plot demonstrating the discrimination between the C1, T1, and T2 groups within each intestinal segment. The clear separation among groups indicates distinct metabolic profiles. (**C**) Permutation test results (n = 200 permutations) validating the robustness and predictive ability of the OPLS-DA models. The original R^2^Y (green) and Q^2^ (blue) values are positioned to the right, with all permuted R^2^Y and Q^2^ values to the left being lower, indicating that the models are not overfitted and possess high predictive capability (Q^2^ > 0.97 for all models).

The principal component analysis (PCA) score plots for the jejunal, cecal, and colonic samples are illustrated in Figure 5A. The evident separation among groups and the close clustering of triplicate samples within each group indicate substantial differences in overall metabolic profiles across the treatments. The score plots generated from the orthogonal partial least squares–discriminant analysis (OPLS-DA) model (Figure 5B) further illustrate distinct separations between groups, exhibiting strong intra-group reproducibility. Additionally, permutation testing of the OPLS-DA model (Figure 5C) revealed high values (R^2^Y = 1, Q^2^ > 0.97), thereby confirming the model’s robust discriminative and predictive capabilities.

#### 3.4.3. Screening and Analysis of Differential Metabolites

**Figure 6:**
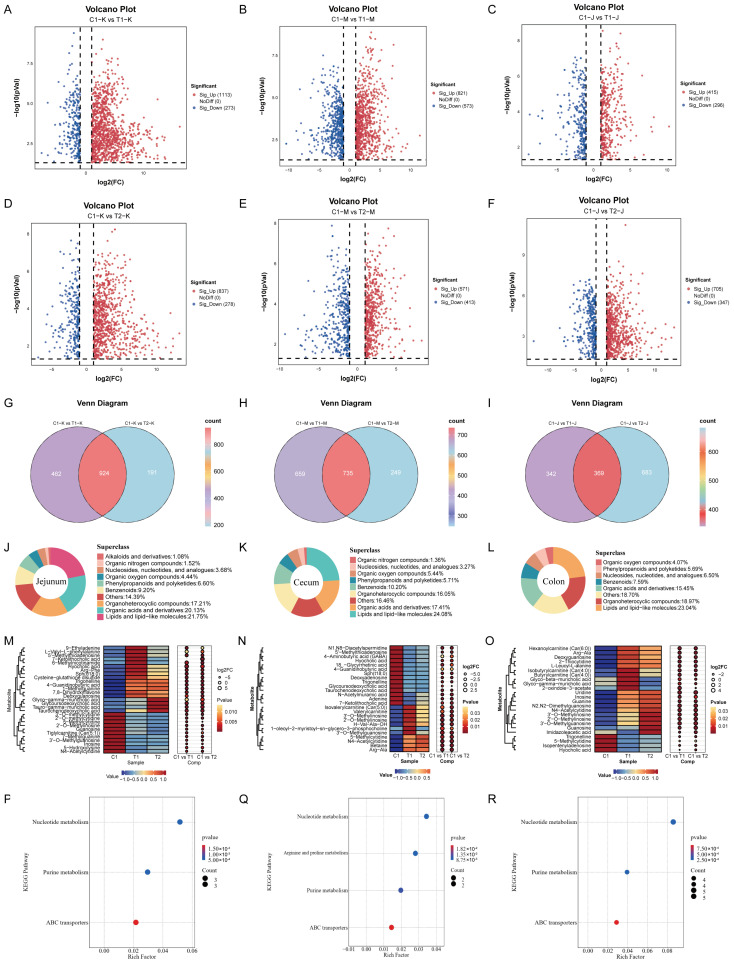
Differential metabolite analysis of jejunal, cecal, and colonic contents in weaned piglets fed different experimental diets (C1, T1, T2). (**A**–**C**) Volcano plots depicting differentially abundant metabolites in the jejunum (**A**), cecum (**B**), and colon (**C**). Red and blue dots represent significantly upregulated and downregulated metabolites, respectively (VIP > 1, FC > 2 or < 0.5, *p* < 0.05). Gray dots indicate metabolites with no significant differences. (**D**–**F**) Venn diagrams showing the numbers of shared and unique differentially abundant metabolites among the three intestinal segments in the T1 vs. C1 (**D**), T2 vs. C1 (**E**), and T1 vs. T2 (**F**) comparisons. (**G**–**I**) Classification statistics of the identified differential metabolites in the jejunum (**G**), cecum (**H**), and colon (**I**). Metabolites are categorized based on their chemical classes. (**J**–**O**) Heatmaps illustrating the relative abundance profiles of key differential metabolites across individual samples in the jejunum (**J**,**K**), cecum (**L**,**M**), and colon (**N**,**O**). Rows represent metabolites, columns represent samples, and the color scale indicates normalized abundance (red: high; blue: low). (**P**–**R**) KEGG pathway enrichment analysis of the differential metabolites in the jejunum (**P**), cecum (**Q**), and colon (**R**). The bubble size represents the number of metabolites enriched in each pathway, and the color gradient indicates the *p* value (red: lower *p* value; blue: higher *p* value). Only the top 20 enriched pathways are shown.

Differential metabolites were screened using the criteria of VIP > 1, FC > 2 or <0.5, and *p* < 0.05. Volcano plots (Figure 6A–F) demonstrated that a significant number of differential metabolites were identified in the jejunum, cecum, and colon when comparing each treatment group to the control group, with the number of metabolites ranging from 711 to 1394. Of these, upregulated metabolites accounted for 62.5–71.3% across all comparisons, while downregulated metabolites represented 28.7–37.5%. The majority of which were upregulated. Further analysis of common differential metabolites across the groups (Figure 6G–I) revealed 924, 735, and 369 metabolites in the jejunum, cecum, and colon, respectively. Classification statistics (Figure 6J–L) indicated that these shared metabolites predominantly belonged to the categories of lipids and lipid-like molecules, organic acids and derivatives, and organoheterocyclic compounds.

### 3.5. Correlation Analysis Between Predominant Intestinal Microbiota and Differential Metabolites

**Figure 7:**
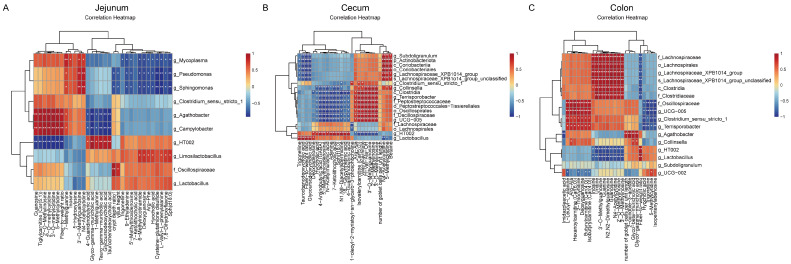
Spearman correlation analysis between key differential microbial taxa and metabolites in different intestinal segments of weaned piglets. The heatmap displays correlation coefficients, with red indicating positive correlations and blue indicating negative correlations. Significant correlations are marked with asterisks (* *p* < 0.05; ** *p* < 0.01). (**A**) Jejunum. (**B**) Cecum. (**C**) Colon.

Spearman correlation analysis was conducted to evaluate the relationships between microbiota and metabolites across each intestinal segment (Figure 6). In the jejunum (Figure 7A), *Oscillospiraceae* exhibited a positive correlation with the villus height-to-crypt depth ratio. *Limosilactobacillus* demonstrated positive correlations with various metabolites, including 6-methylnicotinamide and hyocholic acid, while *Mycoplasma* and other genera showed negative correlations with these metabolites. Additionally, g_HT002 was positively correlated with bile acids such as glycoursodeoxycholic acid but negatively correlated with several methylated nucleotides and the feed-to-gain ratio (F/G). In the cecum (Figure 7B), *Lactobacillus* was positively correlated with taurochenodeoxycholic acid and the F/G. g_HT002 exhibited broad positive correlations with metabolites, including γ-aminobutyric acid (GABA) and N^1^,N^8^-diacetylspermidine, whereas *Clostridium* and several other microbial taxa displayed contrasting association patterns. In the colon (Figure 7C), both *Lactobacillus* and g_HT002 were positively correlated with the F/G but negatively correlated with nucleosides such as inosine and guanosine. *Oscillospiraceae* and related taxa were positively correlated with various acylcarnitines (such as hexanoylcarnitine). *Alloprevotella* demonstrated positive correlations with specific bile acids and the number of goblet cells. Multiple pairs of highly significant correlations were identified across all intestinal segments.

## 4. Discussion

This study integrated 16S rDNA high-throughput sequencing with untargeted metabolomics to jointly analyze the gut microbiota and luminal metabolites in the jejunum, cecum, and colon of nursery pigs. It systematically evaluated the effects of different experimental diets (T1 and T2 groups) on the intestinal microbial structure and metabolome of nursery pigs and explored the potential links between microbiota and metabolites.

### 4.1. Compound Microbial Fermented Feed Significantly Alters Gut Microbiota Structure and Diversity

Alpha diversity analysis revealed that the T2 group significantly reduced the Chao1 index in the cecal microbiota. The Shannon index was significantly decreased in the cecal and colonic microbiota of the T1 group and in the jejunal and cecal microbiota of the T2 group. These changes in alpha diversity are consistent with previous studies showing that fermented feed can reduce microbial diversity in weaned piglets [5,6,9]. The more pronounced reduction in the T2 group suggests a dose-dependent effect, as also observed by Ji et al. [5]. Beta diversity analysis (PCoA) further confirmed distinct separation in the microbial community structure of the jejunum, cecum, and colon among the groups, demonstrating that the probiotic-fermented feed significantly reshaped the gut microbial ecosystem. Similar restructuring of gut microbiota by fermented feed has been reported in piglets [7,13]. Linear discriminant analysis effect size (LEfSe) identified bacterial taxa with significantly different relative abundances at multiple taxonomic levels (phylum, family, genus) between the T1, T2, and C1 groups in all three intestinal segments. For instance, the T1 group showed enrichment of Oscillospiraceae in the jejunum; *Clostridia*, *Oscillospiraceae*, and g_UCG-005 in the cecum; and g_UCG-005, *Oscillospiraceae*, and *Agathobacter* in the colon. Enrichment of *Oscillospiraceae*, a butyrate-producing family associated with improved gut health, has been linked to probiotic supplementation in pigs [13,14]. The T2 group was enriched with g_HT002 in the jejunum; *Lachnospiraceae*_XPB1014_group and *Actinobacteriota* in the cecum; and *Lachnospiraceae* and *Clostridiaceae* in the colon. Increased abundance of *Lachnospiraceae* is often observed with beneficial dietary interventions and is associated with SCFA production [14,15]. The C1 group was generally enriched with *Bacteroidota*, *Prevotellaceae*, *Lactobacillus*, and *Ruminococcaceae*, among others. The higher abundance of Lactobacillus in controls may reflect the typical microbiota of pigs fed a standard corn–soybean meal diet, as reported previously [10,12].

### 4.2. Intestinal Metabolites Are Significantly Altered and Correlated with Changes in Microbiota Structure

Metabolomic analysis identified a total of 2941 metabolites, predominantly classified as lipids and lipid-like molecules, organic acids and derivatives, and organoheterocyclic compounds. Principal component analysis (PCA) and the orthogonal partial least squares–discriminant analysis (OPLS-DA) model revealed clear separations in the metabolic profiles of jejunal, cecal, and colonic contents among the groups, with the model demonstrating high discriminatory and predictive capability (Q^2^ > 0.97). Differential metabolite analysis indicated that a substantial number of metabolites changed significantly in all treatment groups compared to the control, with the most pronounced changes occurring in the jejunum and cecum. KEGG enrichment analysis showed that the differential metabolites were primarily enriched in pathways such as nucleotide metabolism, purine metabolism, ABC transporters, and arginine and proline metabolism. These pathways are known to be influenced by probiotics and fermented feeds, affecting energy metabolism and intestinal health [17,18].

Notably, this study found segment-specific reprogramming of bile acid metabolism. For example, various bile acids like hyocholic acid, glycoursodeoxycholic acid, and taurochenodeoxycholic acid were significantly upregulated in the jejunum but downregulated in the cecum (such as hyocholic acid, glycoursodeoxycholic acid, and taurochenodeoxycholic acid) and colon (such as hyocholic acid). This suggests that feeding probiotic-fermented feed may affect bile acid metabolism, enterohepatic circulation, and microbiota-host interactions. Upregulation in the jejunum could enhance lipid digestibility or signaling, while downregulation in the cecum and colon might indicate altered enterohepatic circulation efficiency or reduced microbial metabolism, potentially helping to lower the load of potentially cytotoxic secondary bile acids in the hindgut.

Previous research indicates that intestinal bile acids play crucial roles in fat digestion and absorption and act as potent signaling molecules for nuclear receptors like the farnesoid X receptor (FXR) and the membrane-bound G protein-coupled bile acid receptor-1 (GPBAR-1). They are involved in maintaining intestinal barrier integrity, gene expression, metabolic homeostasis, and the structure and function of the microbiota [16]. Previous study [18] found that the probiotic mixture promoted ileal bile acid deconjugation and subsequent fecal bile acid excretion by downregulating the intestinal-hepatic FXR-FGF15 axis, thereby inducing hepatic bile acid neosynthesis. Some research [19] demonstrated, through both metagenomic data and fecal microbiota transplantation models, that reductions in secondary bile acids depended on the action of the gut microbiota. Our study observed a marked increase in the *Firmicutes*-to-*Bacteroidetes* (F/B) ratio in the jejunum of the T1 and T2 groups. Studies suggest that an increased F/B ratio is indicative of promoted lipid accumulation [20], which aligns with the observed upregulation of various bile acids in the jejunum.

Significant upregulation of various carnitine species (such as isovaleryl-L-carnitine, and valerylcarnitine) in the cecum and others (such as hexanoylcarnitine, butyrylcarnitine, and isobutyrylcarnitine) in the colon suggests enhanced fatty acid β-oxidation metabolism. Recent findings indicate that carnitine and acylcarnitines function not merely as cofactors or metabolites of fatty acid oxidation but play more complex and multifaceted roles in energy metabolism, mitochondrial homeostasis, epigenetic regulation, inflammation and immunomodulation, tumor biology, signal transduction, and neuroprotection [21]. Changes in various nucleosides and their derivatives in the cecum and colon are consistent with the enriched nucleotide metabolism pathways, hinting that feeding fermented feed may affect nucleotide synthesis and degradation, potentially linked to rapid intestinal epithelial renewal or alterations in gut microbiota structure and function. Research has shown that nucleotides play an important role in regulating the intestinal barrier in piglets by improving intestinal morphology, such as increasing the villus height-to-crypt depth ratio [22]. Liu [23] found that dietary nucleotide supplementation in very early-weaned piglets helped modulate gut microbiota structure. Examination of specific metabolite changes with directionality revealed segment-specific metabolic reprogramming following fermented feed supplementation. In the jejunum, conjugated bile acids, including hyocholic acid, glycoursodeoxycholic acid (GUDCA), and taurochenodeoxycholic acid (TCDCA), were significantly upregulated in the T1 and T2 groups (Figure 5A). This upregulation likely enhances dietary lipid digestion and absorption, contributing to the improved feed efficiency (reduced F/G) observed in treatment groups, while also potentially strengthening intestinal barrier function via FXR/TGR5 signaling. Conversely, in the cecum and colon, the same bile acids were significantly downregulated (Figure 5B,C), suggesting reduced microbial deconversion to potentially cytotoxic secondary bile acids and supporting the enhanced colonic epithelial integrity (increased goblet cells) observed in the T1 group. Regarding energy metabolism, various acylcarnitines (e.g., isovaleryl-L-carnitine, hexanoylcarnitine) were significantly upregulated in the cecum and colon (Figure 5B,C), indicating enhanced fatty acid β-oxidation to provide energy for intestinal epithelial renewal. Additionally, nucleosides, including inosine and uridine, were downregulated in the colon (Figure 5C), potentially reflecting increased utilization by host epithelium for regeneration or by gut microbiota as a nitrogen source. These direction-specific metabolite changes provide mechanistic links between the dietary intervention and the observed improvements in growth performance and intestinal health. In summary, the metabolomics data suggest that feeding probiotic-fermented feed may comprehensively improve the intestinal functional status of nursery pigs by coordinately regulating host bile acid cycling, energy metabolism, and nucleotide metabolism, potentially influencing gut microbiota structure through altered intestinal metabolites [24,25].

### 4.3. Microbiota-Metabolite Correlation Analysis Reveals Potential Microbial Metabolic Functions

Spearman correlation analysis between dominant microbial taxa and key differential metabolites across the jejunum, cecum, and colon revealed intricate inter-kingdom associations, suggesting that gut microbiota modulate the metabolic status of nursery pigs through distinct site-specific activities.

In the jejunum, the primary site for nutrient absorption, identified correlations underscore functional roles in nutrient acquisition, mucosal barrier maintenance, and immune regulation. *Oscillospiraceae* abundance was positively correlated with villus height and the villus height-to-crypt depth ratio. Given that *Oscillospiraceae* are recognized producers of short-chain fatty acids (SCFAs) that fuel epithelial cells and reinforce barrier integrity [26,27], this taxon likely supports small intestinal absorptive capacity via metabolite-mediated signaling. *Limosilactobacillus*, which facilitates carbohydrate fermentation and acidification, exhibited positive correlations with various neuroactive and bioactive metabolites—including the GABA precursor 6-methylnicotinamide, the anti-inflammatory bile acid hyocholic acid, the neuroprotective agent 7,8-dihydroxyflavone, and the antioxidant dipeptide L-valyl-L-phenylalanine. This suggests a multifaceted role for Limosilactobacillus in enhancing jejunal mucosal health and local antioxidant defenses. Notably, these metabolic shifts correlate with the observed depletion of potential pathogens, such as *Campylobacterota*, *Mycoplasma*, and *Pseudomonas*, in the treatment groups. Furthermore, the genus g_HT002 was positively associated with conjugated bile acids (e.g., taurine- and glycine-conjugated species) and 4-guanidinobutyric acid, implying a role in bile acid homeostasis—a critical process for lipid absorption and local signaling in the proximal intestine [28,29,30,31,32,33]. The correlation between g_HT002 and bile acid profiles, alongside its negative association with the feed-to-gain ratio (F/G), suggests that g_HT002 may serve as a beneficial metabolic node, though its functional impact appears highly niche-dependent, as evidenced by the inversion of these associations in the distal gut.

In the cecum, g_HT002 was positively correlated with γ-aminobutyric acid (GABA), N-acetylmuramic acid, and N^1^,N^8^-diacetylspermidine. The latter, a polyamine metabolite essential for cell proliferation [33], highlights the potential role of g_HT002 in regulating systemic signaling. We observed divergent metabolic patterns between g_HT002 and other taxa, such as *Clostridium*_sensu_stricto_1 and g_UCG-005, suggesting the existence of distinct functional modules: one focused on neuro-immunomodulation and another on fiber degradation and energy harvesting. This reinforces the status of g_HT002 as a versatile, keystone bacterium influencing host growth.

The colon, primarily responsible for water resorption and fecal formation, exhibited a distinct microbial-metabolic landscape. Lactobacillus and g_HT002 were positively correlated with F/G but inversely associated with nucleosides (e.g., inosine, uridine). Given that nucleoside levels reflect cellular energy status and purine salvage pathway activity, these taxa may indirectly influence host purine metabolism. Furthermore, we identified a coupling between Oscillospiraceae, UCG-005, and Terrisporobacter with acylcarnitines—intermediates of fatty acid β-oxidation [34,35,36,37,38,39]—suggesting that the colonic microbiota may be tuned to the host’s mitochondrial energy state. Notably, the genus Agathobacter was positively correlated with tryptophan-derived 2-hydroxyindole-3-acetate, glycohyocholic acid, and goblet cell density. As a canonical butyrate producer [36], Agathobacter likely promotes goblet cell proliferation and mucin secretion via WNT/ERK signaling [37], while its tryptophan metabolism potentially generates aryl hydrocarbon receptor (AhR) ligands, thereby reinforcing the colonic barrier [38,39]. These findings elucidate the “microbiota-metabolite-host” axis, identifying candidate microbial targets for functional validation. Future research integrating transcriptomics or proteomics will be essential to delineate the precise regulatory circuits governing these metabolic pathways and to translate these insights into targeted nutritional interventions for swine health.

## 5. Conclusions

In conclusion, dietary supplementation with compound microbial fermented feed significantly enhanced the growth performance and intestinal health of weaned piglets. These benefits were facilitated by the restructuring of gut microbiota, specifically an enrichment of *Oscillospiraceae* and *Lachnospiraceae*, along with the modulation of nucleotide and bile acid metabolism. Notably, the group with a 50% replacement (T1) demonstrated superior improvements in intestinal morphology and achieved a more balanced microbial-metabolic profile. Therefore, it is recommended that a 50% replacement of the basal diet with compound microbial fermented feed be adopted as the optimal inclusion level for weaned piglets.

## Figures and Tables

**Table 1:** Calculated and analyzed nutrient composition of the experimental diets for nursery pigs (%, as dry matter basis).

Items	C1	T1	T2
Ingredient			
Corn	65.50	32.75	0.00
Soybean meal (43% CP)	22.00	11.00	0.00
Fermented corn	0.00	32.75	65.50
Fermented soybean meal	0.00	11.00	22.00
Fish meal	3.00	3.00	3.00
Soybean oil	2.50	2.50	2.50
Whey powder	3.00	3.00	3.00
Limestone	0.80	0.80	0.80
Dicalcium phosphate	0.60	0.60	0.60
L-Lysine HCl (78%)	0.30	0.30	0.30
DL-Methionine (99%)	0.10	0.10	0.10
L-Threonine (98%)	0.05	0.05	0.05
Salt	0.30	0.30	0.30
Premix ^1^	1.85	1.85	1.85
Total	100	100	100
Calculated nutrient levels			
ME (MJ/kg) ^2^	14.23	14.23	14.23
Crude protein (CP, %)	18.00	18.00	18.00
Ether extract (EE, %)	4.50	4.50	4.50
Crude fiber (CF, %)	2.20	2.30	2.40
Ash (%)	5.20	5.25	5.35
Calcium (Ca, %)	0.70	0.70	0.70
Total phosphorus (P, %)	0.60	0.60	0.60
Available P (%)	0.40	0.40	0.40
SID Lysine (%) ^3^	1.15	1.15	1.15
SID Methionine (%)	0.36	0.36	0.36
SID Threonine (%)	0.68	0.68	0.68
Determined nutrient levels ^4^			
Gross energy (GE, MJ/kg)	16.85	16.90	17.02
Crude protein (CP, %)	17.88	18.05	18.23
Ether extract (EE, %)	4.42	4.55	4.61
Neutral detergent fiber (NDF, %)	9.50	9.80	10.20
Acid detergent fiber (ADF, %)	3.80	4.00	4.30
Ash (%)	5.20	5.25	5.35
Calcium (Ca, %)	0.71	0.69	0.70
Total phosphorus (P, %)	0.59	0.61	0.60
SID Lysine (%)	1.13	1.14	1.14
SID Methionine (%)	0.35	0.36	0.35
SID Threonine (%)	0.67	0.68	0.67

^1^ Premix provided per kilogram of complete diet: vitamin A 80,000 IU, vitamin D_3_ 20,000 IU, vitamin E 150 IU, vitamin B_2_ 40 mg, pantothenic acid 100 mg, niacinamide 120 mg, Fe (as ferrous sulfate) 1200 mg, Cu (as copper sulfate) 150 mg, Mn (as manganese sulfate) 400 mg, Zn (as zinc sulfate) 800 mg, I (as calcium iodate) 0.8 mg, Se (as sodium selenite) 0.3 mg. ^2^ ME, metabolizable energy (converted from Mcal/kg using 1 Mcal = 4.184 MJ). ^3^ SID, standardized ileal digestible amino acid levels were calculated based on analyzed total amino acid concentrations and published standardized ileal digestibility coefficients. ^4^ Determined nutrient levels were obtained from chemical analysis of the experimental diets. C1: control group (basal diet); T1: 50% fermented feed group; T2: 100% fermented feed group.

**Table 2:** Effects of compound microbial fermented feed on the growth performance of piglets.

Items	C1	T1	T2	*p* Value
Initial weight, kg/pig	11.55 ± 0.11	12.33 ± 0.19	11.96 ± 0.14	0.557
Final weight, kg/pig	25.32 ± 5.80 ^c^	28.77 ± 2.22 ^b^	30.97 ± 4.95 ^a^	0.031
ADG, kg/(pig·d)	0.41 ± 0.23 ^b^	0.50 ± 0.53 ^a^	0.57 ± 0.55 ^a^	0.047
ADFI, kg/(pig·d)	1.08 ± 0.06	0.93 ± 0.10	0.84 ± 0.12	0.078
F/G ratio	2.61 ± 0.13 ^a^	1.88 ± 0.21 ^b^	1.47 ± 0.13 ^c^	0.029

C1: Control group (basal diet); T1: 50% fermented feed group; T2: 100% fermented feed group (n = 6) ADG, average daily gain; ADFI, average daily feed intake; F/G, feed-to-gain ratio. Note: different lowercase letters within the same row indicate significant differences (*p* < 0.05), while the same letters indicate no significant difference (*p* > 0.05).

**Table 3:** Effects of compound microbial fermented feed on the intestinal morphology and structure of nursery pigs.

Segment	Items	C1	T1	T2	*p* Value
Jejunum	Villus height (VH, mm)	0.32 + 0.11 ^ab^	0.45 + 0.03 ^a^	0.39 ± 0.02 ^b^	0.028
	Crypt depth (CD, mm)	0.32 ± 0.08 ^a^	0.34 ± 0.03 ^a^	0.38 ± 0.03 ^a^	0.350
	VH/CD ratio	0.96 ± 0.12 ^b^	1.32 ± 0.02 ^a^	0.80 ± 0.06 ^b^	0.017
Cecum	Goblet cells (count)	26.33 ± 4.51 ^b^	56.67 ± 4.62 ^a^	61.00 ± 1.00 ^a^	0.039
	Epithelial length (mm)	0.45 ± 0.01 ^b^	0.92 ± 0.02 ^a^	0.90 ± 0.20 ^a^	0.001
	Goblet cells per unit length (count/mm)	58.26 ± 8.15 ^a^	61.52 ± 5.36 ^a^	70.11 ± 16.19 ^a^	0.088
Colon	Goblet cells (count)	33.33 ± 5.86 ^b^	67.00 ± 7.94 ^a^	34.33 ± 4.51 ^b^	0.001
	Epithelial length (mm)	0.54 ± 0.06 ^c^	0.86 ± 0.02 ^a^	0.68 ± 0.04 ^b^	0.026
	Goblet cells per unit length (count/mm)	62.08 ± 5.29 ^b^	78.03 ± 7.82 ^a^	50.49 ± 7.62 ^b^	0.011

Note: Different lowercase letters within the same row indicate significant differences (*p* < 0.05), while the same letters indicate no significant difference (*p* > 0.05), (n = 6).

## Data Availability

The original contributions presented in this study are included in the article/Supplementary Materials. Further inquiries can be directed to the corresponding author(s).

## Supplementary Materials

The following supporting information can be downloaded at https://www.mdpi.com/article/10.3390/ani16060972/s1. Table S1. Recommended dietary nutrient specifications for nursery pigs (11–25 kg body weight) with varying inclusion levels of fermented feed; Table S2. Calculated and analyzed nutrient composition of the experimental diets for nursery pigs (%, as dry matter basis).

## Abbreviations

The following abbreviations are used in this manuscript:

ADG	Average daily gain
ADFI	Average daily feed intake
F/G	Feed-to-gain ratio
LC-MS	Liquid chromatography-mass spectrometry
UPLC	Ultra-performance liquid chromatography
HE	Hematoxylin–eosin
AB-PAS	Alcian Blue–Periodic Acid Schiff
ASV	Amplicon sequence variant
LEfSe	Linear discriminant analysis effect size
PCA	Principal component analysis
OPLS-DA	Orthogonal partial least squares–discriminant analysis
VIP	Variable Importance in Projection
FC	Fold change
KEGG	Kyoto Encyclopedia of Genes and Genomes
GABA	γ-Aminobutyric acid
F/B	Firmicutes-to-Bacteroidetes ratio
FXR	Farnesoid X receptor
GPBAR-1	G protein-coupled bile acid receptor-1
FGF15	Fibroblast growth factor 15
ATP	Adenosine triphosphate
ADP	Adenosine diphosphate
AMP	Adenosine monophosphate
AhR	Aryl hydrocarbon receptor
WNT	Wingless-related integration site
ERK	Extracellular signal-regulated kinase
SCFA	Short-chain fatty acid
DNA	Deoxyribonucleic acid
PCR	Polymerase chain reaction
rRNA	Ribosomal RNA
VH	Villus height
CD	Crypt depth
VH/CD	Villus height-to-crypt depth ratio
SD	Standard deviation
ANOVA	Analysis of Variance
NRC	National Research Council
ARRIVE	Animal Research: Reporting of In Vivo Experiments
