# Supplementary material for: Composite Probiotic Fermented Feed Enhances Growth Performance and Intestinal Health in Weaned Piglets by Modulating the Gut Microbiome and Metabolome
Source: Animals (Basel). 2026 Mar 20;16(6):972. doi: 10.3390/ani16060972 (PMC13023348; doi:10.3390/ani16060972)
Supplement: Supplementary file 1 [file animals-16-00972-s001.zip › animals-4172662-supplementary.pdf]

Table S1. Recommended dietary nutrient specifications for nursery pigs (11-25 kg body weight) with varying inclusion levels of fermented feed.

Nutrient Item	Unit	C1	T7	T8
**Conventional Components**				
Metabolizable Energy (ME)	MJ/kg	13.8 – 14.2	13.5 – 13.9	13.2 – 13.7
Crude Protein (CP)	%	18.0 – 20.0	17.5 – 19.0	17.0 – 18.5
Standardized Ileal Digestible Lysine (SID Lys)	%	1.25 – 1.35	1.20 – 1.30	1.15 – 1.25
Calcium (Ca)	%	0.70 – 0.80	0.70 – 0.80	0.70 – 0.80
Total Phosphorus (TP)	%	0.60 – 0.65	0.60 – 0.65	0.60 – 0.65
Available Phosphorus (AP)	%	0.35 – 0.40	0.33 – 0.38	0.30 – 0.35
Crude Fiber (CF)	%	< 3.0	< 3.5	< 4.0
**Key Additive Adjustments**				
External Acidifier	%	0.3 – 0.5	0 – 0.2	0

Table S2. Calculated and analyzed nutrient composition of the experimental diets for nursery pigs (%, as dry matter basis).

Items	C1	T7	T8
**Ingredient**			
Corn	65.50	31.9	0.00
Soybean meal (43% CP)	22.00	11.00	0.00
Fermented Corn	0.00	31.90	62.10
Fermented Soybean meal	0.00	11.00	22.00
Fish meal	3.00	3.00	3.00
Soybean oil	2.50	2.50	2.50
Whey powder	3.00	3.00	3.00
Limestone	0.80	0.80	0.80

Items	C1	T7	T8
Dicalcium phosphate	0.60	0.60	0.60
L-Lysine HCl (78%)	0.30	0.30	0.30
DL-Methionine (99%)	0.10	0.10	0.10
L-Threonine (98%)	0.05	0.05	0.05
Salt	0.30	0.30	0.30
**Premix**	**1.85**	**1.85**	**1.85**
**Total**	**100**	**100**	**100**
**Calculated nutrient levels**			
ME, Mcal/kg	3.40	3.40	3.40
CP	18.00	18.00	18.00
EE	4.50	4.50	4.50
CF	2.20	2.30	2.40
Ca	0.70	0.70	0.70
Total P	0.60	0.60	0.60
Available P	0.40	0.40	0.40
SID Lys	1.15	1.15	1.15
SID Met	0.36	0.36	0.36
SID Thr	0.68	0.68	0.68
**Determined nutrient levels**			
GE, MJ/kg	16.85	16.90	17.02
CP	17.88	18.05	18.23
EE	4.42	4.55	4.61
NDF	9.50	9.80	10.20
ADF	3.80	4.00	4.30
Ash	5.20	5.25	5.35

Premix: Provided per kilogram of complete diet: Vitamin A 80,000 IU, Vitamin D_3 20,000 IU, Vitamin E 150 IU, Vitamin B_2 40 mg, Pantothenic acid 100 mg, Niacinamide 120 mg, Fe (as ferrous sulfate) 1,200 mg, Cu (as copper sulfate) 150 mg, Mn (as manganese sulfate) 400 mg, Zn (as zinc sulfate) 800 mg, I (as calcium iodate) 0.8 mg, Se (as sodium selenite) 0.3 mg.ME, metabolizable energy; CP, crude protein; EE, ether extract; CF, crude fiber; SID, standardized ileal digestible; GE, gross energy; NDF, neutral detergent fiber; ADF, acid detergent fiber.The calculation of the nutritional value of all raw materials is based on the recommended data of the "China Feed Ingredients and Nutritional Value Table" (23rd Edition).
